# Reptile- and Amphibian-associated Salmonellosis in Childcare Centers, United States

**DOI:** 10.3201/eid1812.120784

**Published:** 2012-12

**Authors:** Neil M. Vora, Kristine M. Smith, Catherine C. Machalaba, William B. Karesh

**Affiliations:** Author affiliations: Columbia University, New York, New York, USA (N.M. Vora);; EcoHealth Alliance, New York (N.M. Vora, K.M. Smith, C.C. Machalaba, W.B. Karesh)

**Keywords:** amphibian, child care center, childcare, reptile, bacteria, pediatric, salmonellosis, Salmonella, United States

**To the Editor:** *Salmonella* spp. infection represents a major public health problem in the United States; nearly 1.4 million human cases and 600 associated deaths are reported each year (*1*). Reptile and amphibian exposures might cause >70,000 of these cases annually (*2*). Furthermore, children are at increased risk of acquiring *Salmonella* spp. and experiencing severe manifestations of disease (*3**,**4*). Given the increasing popularity of reptiles and amphibians as pets, reptile- and amphibian-associated salmonellosis is a substantial public health concern (*5*).

The public has a generally low level of awareness that *Salmonella* spp. can be acquired from reptiles and amphibians (*6*); a poll conducted by the US Centers for Disease Control and Prevention (CDC) during 2003 showed that as few as 4 of 49 states require pet stores to provide information about salmonellosis to persons purchasing reptiles (*4*). A Food and Drug Administration ban, activated in 1975, on the sale of small turtles subsequently prevented an estimated 100,000 cases of salmonellosis in children each year (*7*). To further reduce the risk of reptile- and amphibian-associated salmonellosis, the CDC has issued recommendations advising that children <5 years of age avoid contact with reptiles and amphibians and that these animals not be kept in childcare centers. The CDC also recommends that all persons wash their hands after handling reptiles and amphibians (*8*).

We reviewed the regulations as of December 2011 for childcare centers in all US states aimed at preventing reptile- and amphibian-associated salmonellosis (Table). To gather these data, we searched the websites for each state’s public health department or the state’s equivalent of an early childhood learning agency. When searches on the Internet did not yield the desired information, the appropriate state agencies were contacted by phone or email. In some instances, we corresponded with the designated State Public Health Veterinarian.

**Table:** State regulations for contact between children and animals in childcare centers, United States, 2011

Description of state regulation	No.(%) states
Bans all animals that show evidence of disease from childcare centers	22 (44)
Bans all potentially dangerous or harmful animals from childcare centers	23 (46)
Bans all reptiles from childcare centers	12 (24)
Bans all amphibians from childcare centers	3 (6)
Requires staff and/or children to wash hands after handling animals	25 (50)

Overall, only 50% of states had regulations that required staff and/or children to wash their hands after touching any animals in childcare centers. Twelve states banned reptiles from childcare centers; 3 of these 12 states also banned amphibians, and these were the only states we found to have banned amphibians from childcare centers. While some states did not allow potentially dangerous or harmful animals in childcare centers, a minority of these states went further to expressly ban reptiles as well (of the 23 states that banned potentially dangerous or harmful animals, 8 states also banned reptiles). One state (Colorado) explicitly banned reptiles, amphibians, and potentially dangerous or harmful animals from childcare centers and also required staff and children in the center to wash their hands after touching animals.

This survey has several limitations. Given the ambiguity in the language used in some regulations and that the language was not standardized between states, we might have misinterpreted some of the documents we reviewed. Furthermore, we might have unintentionally overlooked regulations that were already in place during our investigation, and hence our findings might underestimate the true number of states that have such policies. In some cases, cities and counties have regulations that provide increased protection beyond those implemented at the state level.

In summary, we found great variation between state regulations for childcare centers aimed at reducing transmission of *Salmonella* spp. from reptiles and amphibians to humans. The discrepancy in the regulations of states that banned potentially dangerous or harmful animals from childcare centers but that did not also specifically ban reptiles and amphibians was paradoxical, considering the well-recognized risk that these animals pose for transmitting *Salmonella* spp. We do not know how many childcare centers across the United States currently house reptiles or amphibians. However, our data suggest that there is room for revision of the regulations in many states which could in turn augment efforts to prevent *Salmonella* spp. transmission from reptiles and amphibians. We believe that the recommendations issued by the CDC for the prevention of salmonellosis from reptiles and amphibians (*4*) could serve as a practical guide as state regulations are updated. Our own experience has indicated that greater collaboration between public health organizations and the agencies responsible for setting regulations for childcare centers can be informative and productive. Similarly, state agencies can work with the pet industry and childcare centers to develop approaches that are mutually beneficial.

Although pets provide many benefits to humans, particularly during the early years of life *(**9**)*, any exposure that children have to animals must pose minimal risk to the children’s health. Ultimately, keeping reptiles and amphibians out of childcare centers and requiring that staff and children wash their hands after touching animals offers a simple way to better safeguard the health of children while having a minimal effect on practices of childcare centers.
